# Evaluating the Impact of Intensifying Treatment from Human to Analogue Insulin on Glycaemic Control and Insulin Expenditure in Patients with Type 2 Diabetes: A Retrospective Cohort Study

**DOI:** 10.21315/mjms2024.31.2.14

**Published:** 2024-04-23

**Authors:** Siti Aisyah Hussin, Nur Aimi Mohamad, Mohd Khairi Othman, Wan Mohd Izani Wan Mohamed

**Affiliations:** 1 Department of Internal Medicine, School of Medical Sciences, Universiti Sains Malaysia, Kelantan, Malaysia; 2 Department of Pharmacy, Hospital Universiti Sains Malaysia, Kelantan, Malaysia

**Keywords:** analogue insulin, FBS, HbA1c, human insulin, insulin cost difference, T2DM

## Abstract

**Background:**

Achieving good glycaemic control is essential to reducing the risk of diabetes complications. Insulin is the most effective therapy for achieving good glycaemic control; however, it is associated with a higher risk of hypoglycaemia, especially with human insulin. This study aimed to evaluate the efficacy of intensification from human to analogue insulin and its added cost.

**Methods:**

This retrospective study was conducted at the Hospital Universiti Sains Malaysia (HUSM). Patients with type 2 diabetes mellitus (T2DM) who underwent intensification for at least 3 months from human to analogue insulin were included in this study. The patients’ medical records, haemoglobin A1c (Hba1c) and fasting blood sugar (FBS) were retrieved. The total cost pre- and post-intensification of insulin was obtained from the pharmacy database. Differences in HbA1c, FBS and total insulin cost pre- and post-intensification were analysed.

**Results:**

A total of 163 patients with T2DM who had intensification from human to analogue insulin were included in this study. HbA1c and FBS levels were significantly lower in analogue insulin. However, the differences were not clinically significant, as the mean reduction in HbA1c was less than 0.5%. Meanwhile, the total costs of analogue insulin for 3 months were higher.

**Conclusion:**

There were no clinically significant improvements in patients’ HbA1c and FBS after the intensification of insulin, despite the extra costs spent. Hence, it is vital to choose the right group of patients to receive an insulin analogue to maximise its benefit but at the most optimal cost.

## Introduction

Diabetes mellitus (DM) causes significant morbidity and mortality worldwide, and its impact is likely to increase over the years. Type 1 diabetes mellitus (T1DM) and type 2 diabetes mellitus (T2DM) are different; thus, approaches to treatment differ (1). In Malaysia, it is estimated that 3.9 million (18.3%) of the adult population will have raised blood sugar in 2019, which is expected to increase over the years (2). In 2011, Kelantan recorded the highest number of diabetic patients compared to other states in Malaysia (3) and this is attributable to sugar-laden local delicacies (4).

Analysis of haemoglobin A1c (HbA1c) provides a reliable measure of chronic hyperglycaemia and correlates well with the risk of multiorgan diabetes complications. A reduction in HbA1c by 1% is associated with a significant reduction in microvascular complications by 37% (5, 6). Another study revealed that patients with T2DM with a 1% reduction in HbA1c were associated with a 2% reduction in all-cause total healthcare costs and a 13% reduction in diabetes-related healthcare costs (7). A clinically significant HbA1c decrease is defined as a difference of ≥ 0.5% compared to baseline. This is based on the National Institute for Health and Care Excellence (NICE) guidelines for 2015, according to an analysis by Tyndall et al. in 2019 (8, 9). In Malaysia, a target HbA1c of less than 7% is recommended for the management of T2DM (6th edition) in 2020 (10). The goal of treatment is to achieve good glucose control, avoid multiorgan complications and prevent hypoglycaemic episodes in everyday life. This can be done by mimicking, as closely as possible, the serum level of insulin produced in a healthy person (11).

In low-income countries, human insulin still comprises over 94% of all insulin. In Malaysia, the use of insulin analogues in public hospitals is low, ranging from 2% to 3%. This is mainly due to the price of insulin analogues, which are significantly more expensive (up to three to five times) than human insulin (12). A study by Shafie and Ng (13) showed that in Malaysia, the cost of analogue insulin is higher than that of human insulin, even though the utilisation of insulin in Malaysia is lower than in Australia.

Analogue insulin has been designed to mimic physiologic insulin patterns more closely through improved pharmacokinetic characteristics that result in either more rapid or prolonged pharmacodynamics. Rapid-acting analogue insulin can be administered immediately before eating, producing a rapid and short-lived insulin spike to cater to postprandial glucose rise. This is significantly more convenient for patients compared to human insulin. Long-acting basal insulin analogues have been designed to approach the ideal characteristics of basal insulin by having a relatively flat, 24-h basal insulin supply with less variability in action (11). The use of analogue insulin in Malaysia is still limited, mainly due to its price. Healthcare resources are limited in our country, hence the importance of cost analysis to determine whether insulin analogues are justified for all or some diabetic patients (14).

The total expenditure on insulin at Hospital Universiti Sains Malaysia (HUSM) in 2021 was almost RM4 million, of which over 80% covered the cost of analogue insulin. Therefore, it is crucial to conduct a cost-effective analysis of the use of analogue insulin at HUSM. Previous studies have examined the cost-effectiveness of analogue insulin in various countries.

A study published in the *British Medical Journal* (BMJ) in 2011 (15) assessed the incremental cost of analogue insulin over a 10-year period. The National Health Service (NHS) in United Kingdom (UK) spent a total of £2,732 million on insulin during this period. The annual cost of analogue insulin increased from £18.2 million to £305 million, while the cost of human insulin decreased from £131 million to £51 million. If it were assumed that all patients using analogue insulin could have used human insulin instead, the overall incremental cost of analogue insulin would be £625 million. A systematic review conducted in 2017 (16) suggested that analogue insulin is cost-effective for T1DM but the evidence for its use in T2DM is not convincing.

A Canadian study (17) found that the cost-effectiveness of analogue insulin depended on the type of analogue used and whether the patient had T1DM or T2DM. Insulin aspart, lispro and glargine were found to be more effective, but also more expensive. The incremental costs per quality-adjusted life year were Can$22,488, Can$130,865 and Can$642,099, respectively. Treatment with analogue insulin was associated with a reduction in diabetes-related complications compared to human insulin, but the benefits and cost savings did not offset the increased costs.

A local study in Malaysia by Shafie and Ng in 2020 (13) compared the cost-effectiveness of long-acting analogue insulin with long-acting human insulin. The net cost differences were RM4,868 for insulin glargine and RM6,026 for insulin detemir. The cost savings from preventing severe hypoglycaemia episodes were RM4,377 for insulin glargine and RM12,753 for insulin detemir. The total additional quality-adjusted life years gained were 0.1317 for insulin glargine and 0.8376 for insulin detemir. This indicates that both long-acting analogue insulins are cost-effective for T2DM patients, especially when considering the benefit of reducing hypoglycaemia events.

Another study in Nordic countries (18) assessed the cost-effectiveness of insulin detemir compared to Neutral Protamine Hagedorn (NPH) insulin when initiating insulin treatment for people with T2DM in Denmark, Finland, Norway and Sweden. The lower risk of non-severe hypoglycaemia and less weight gain associated with insulin detemir resulted in economic benefits in the short term. Based on cost/quality-adjusted life year threshold values, this represents a good value for the money in the Nordic countries.

A Health Technology Assessment in the UK in 2004 (19) suggested that insulin glargine effectively reduces the number of nocturnal hypoglycaemic episodes compared to NPH insulin. However, there was no observed improvement in long-term glycaemic control, indicating that insulin glargine is unlikely to reduce the incidence of long-term microvascular and cardiovascular complications of diabetes. According to a Health Technology Assessment report by the Ministry of Health (MOH) Malaysia (14), studies on the incremental cost-effectiveness ratio per quality-adjusted life year generally indicated that insulin analogues could be cost-effective compared to human insulin. Although drug costs were higher for insulin analogues, the reduced complication costs partly offset this.

A comparative analysis of human insulin versus analogue insulin was conducted in a low-income population in 2023 (20). The study revealed that analogue insulin delivered in vials had poorer adherence, higher rates of emergency department visits and hospitalisations, and was less cost-effective compared to human insulin. Users of analogue insulin via pen devices showed better adherence, and this type of insulin was more cost-effective. A pharmacoeconomic study conducted in Brazil in 2017 (21) concluded that human insulins were the best treatment option for diabetes mellitus based on direct cost analysis.

No similar study had been done in Malaysia before, where the glycaemic control and cost implications of insulin intensification from human insulin to analogue insulin were evaluated for a 3-month duration. This study evaluated the past practice of insulin use in HUSM, specifically. Hopefully, the data will help clinicians effectively utilise insulin analogues for the management of T2DM patients. This study will have both practical and financial implications regarding hospital costs for future improvements where resources are finite.

## Methods

### Study Population and Study Design

This retrospective study was conducted at the medical outpatient clinic at HUSM. Patients with T2DM who were on analogue insulin were identified from the outpatient pharmacy database from 2010 to 2020. From this database, patients who had undergone intensification for at least 3 months from human to analogue insulin were included in the study. The inclusion criteria were those aged 18 years old and above who had previously been on human insulin for at least 3 months, then switched to analogue insulin for at least 3 months, diagnosed with T2DM at least 1 year before inclusion in the study, conversion from human insulin to analogue insulin within the same regime (basal, premixed, basal-bolus and bolus) and also on stable other diabetic medications after intensification to analogue insulin. The exclusion criteria were any emergency hospitalisation episode 6 months before and after the change in insulin and significant changes in physical activity, dietary habits and insulin compliance.

The patient’s medical records and investigation results, haemoglobin A1c (Hba1c) and fasting blood sugar (FBS) were retrieved from the Prescription On-Line System (POLS), online discharge summary and Laboratory Information System (LIS). The total cost pre- and post-intensification of insulin was calculated using insulin pricing obtained from the pharmacy using the Drug Pharmacy Inventory Management System (SPIFU). The changes in HbA1c after intensification of human insulin to analogue insulin were analysed, and the results were categorised based on different insulin regimes. A clinically significant change in HbA1c value was defined as a reduction of at least 0.5% from baseline, and the FBS value was targeted to be within a normal range (4.4 mmol/–7 mmol/L). The cost of human and analogue insulin was calculated for 3 months for each patient based on the insulin price per unit and the total insulin dosage used per day.

### Statistical Analysis

All statistical analyses were performed with IBM^®^ Statistical Package for Social Science (SPSS^®^) version 26.0 software (IBM^®^, Armonk, New York, United States). Categorical variables were presented as frequency and percentage, whereas numerical variables were presented as mean (standard deviation [SD]) for normally distributed variables. Paired *t*-tests were used to compare mean HbA1c, FBS value and the 3-month total cost change pre- and post-intensification from human insulin to analogue insulin. The statistical significance level was set as *P*-value; values of < 0.05 were reported.

## Results

A total of 1,818 T2DM patients on human insulin, then intensified to analogue insulin from 2010 to 2020 in HUSM, were screened. Out of that, 1,025 patients were excluded due to incomplete data on glycaemic control (HbA1c and/or FBS). Only 163 patients fulfilled the inclusion and exclusion criteria. Thus, 163 patients with T2DM who had an intensification from human insulin to analogue insulin at HUSM were included in this study. This consisted of 80 (49.1%) males and 83 (50.9%) females. The ages of the patients ranged from 27 years old to 75 years old, with a mean age of 57.87 (SD 10.20) years old. Most of the patients were aged 41 years old–80 years old (95%). Out of 163 patients, 51 (31.3%) had a basal insulin regime, 72 (44.2%) had a premixed insulin regime, 16 (9.8%) had a basal bolus insulin regime, and 24 (14.7%) had a bolus insulin regime (Table 1).

Table 2 shows the differences in glycaemic controls for HbA1c and FBS before and after intensification from human insulin to analogue insulin. Both HbA1c and FBS levels were significantly lower in analogue insulin compared to human insulin. Table 3 shows the differences in HbA1c and FBS for four different insulin regimes (basal, premixed, basal bolus and bolus) before and after intensification from human insulin to analogue insulin. Only the premixed insulin regimen and bolus insulin regimen had significantly lower HbA1c levels in analogue insulin compared to human insulin. Meanwhile, there was significantly lower FBS in analogue insulin compared to human insulin for the basal insulin regimen. In terms of cost, 3 months’ usage of analogue insulin recorded a significantly higher cost compared to human insulin (Table 4).

## Discussion

In this study, most of the patients were aged 41 years old and above, as this study included only T2DM patients (95%). In terms of gender, the proportion of male and female patients was similar, as reported in the National Diabetes Registry Report 2013 to 2019 (2). Most of the patients included in this study were on a premixed insulin regime, where most of them were switched from biphasic isophane insulin to novomix insulin (44.2%).

A clinically significant HbA1c decrease was defined as a difference of ≥ 0.5% from the baseline. This is based on the UK’s NICE guidelines for 2015, according to an analysis by Tyndall et al. in 2019 (8, 9). A reduction of HbA1c by 1% is associated with a significant reduction of microvascular complications by 37%, based on two large-scale studies, the UK Prospective Diabetes Study 1998 in T2DM and the UK Diabetes Control and Complications Trial 1993 in T1DM (5, 6). The target of HbA1c should be individualised based on patients’ profiles, where most patients are targeted for HbA1c of less than 7%, as suggested by the 6th edition of the Clinical Practice Guideline (CPG) 2020 (10).

The mean HbA1c post-intensification was still above the target HbA1c (9.67%), meaning that the patients were still at a high risk of developing microvascular and macrovascular complications of T2DM. This study showed a mean HbA1c reduction of 0.45% (*P*-value < 0.05) after intensification to analogue insulin. This reduction did not meet the standard criteria suggested in multiple guidelines to reduce the risk of DM-related complications, despite the significant cost difference between human and analogue insulin. This result is consistent with other studies in the past that measured the mean difference in HbA1c. This might be due to other confounding factors, such as poor injection technique, medication adherence and intolerance towards the intensification of insulin treatment.

In terms of the mean change in FBS, there was a statistically significant drop after intensification from human insulin to analogue insulin. Based on our CPG 2020, 6th edition (10), the target FBS for patients on treatment should be between 4.4 mmol/L and 7 mmol/L. Our results showed that, despite a decrease in FBS levels, the mean value was far from our target (9.25 mmol/L).

Further evaluation of the mean change in HbA1c and FBS for the four different regimes found that only the premixed insulin regimen and bolus insulin regimen had significantly lower HbA1c in analogue insulin compared to human insulin. This result contrasts with previous studies, as stated in a systemic review, in which premixed insulin analogues appeared to be similar in lowering HbA1c (22). The findings of two other randomised controlled trials showed that HbA1c control was the same in both human and analogue insulin for the premixed regime (23, 24). For short-acting insulin, the Health Technology Assessment (HTA) reported no statistically significant differences between insulin lispro and regular human insulin treatments (12). Likewise, another study reported that the HbA1c from a pooled analysis of 11 trials involving 3,093 patients was only −0.03% (−0.12–0.06%) for insulin lispro (25). Insulin aspart also did not result in a significant reduction in HbA1c levels compared with human insulin, as demonstrated by a pooled analysis of studies based on three systematic reviews (12, 25, 26). This difference could be explained by the difference in our study population’s demography compared to previous studies and due to our small sample size.

Based on this study, it was found that there was no significant difference in HbA1c observed in the basal insulin regimen and basal bolus insulin regimen between human insulin and analogue insulin. This is in keeping with many previous studies that have been conducted in the past. A study showed that there was no evidence that insulin glargine was more effective than NPH insulin in reducing either FBS or HbA1c and some evidence that both insulins were as effective as each other in both FBS and HbA1c controls (26). In a randomised, open-label, two-way, cross-over study among T2DM patients treated with insulin glargine versus NPH insulin, it was reported that both insulin glargine and NPH insulin provided similar improvements in terms of glycaemic control (27). Two HTA reports (28, 29) and two systematic reviews (25, 30) reported similar HbA1c values for T2DM patients treated with insulin detemir compared to those treated with NPH insulin.

In this study, it was evident that there was a statistically significant difference in the mean 3-month cost amounts for human insulin compared to analogue insulin, with a mean difference of RM 292.85 (95% CI: 265.79, 319.91). Another noteworthy finding in this study was that, even though the intensification from human insulin to analogue insulin only resulted in minimal changes to HbA1c and FBS, it resulted in dramatic increases to the insulin costs. The mean values for both HbA1c and FBS were also far from the optimal values or the target values needed to ensure a significant reduction in T2DM microvascular and macrovascular complications. However, this is a crude or simple cost-change calculation for three months of use of insulin and not an extensive cost analysis, and costs for hospitalisation due to hypoglycaemia and T2DM-related complications were not included in this study. This was the first study in Malaysia to focus on the glycaemic control and cost implications of insulin intensification from human insulin to analogue insulin during an evaluation period of 3 months. The use of insulin analogues has been found to help patients avoid hypoglycaemia and better adhere to their insulin treatments, which is why their value is worth serious consideration that extends beyond a focus on their price. This is based on modelling for cost-effectiveness analyses and the willingness-to-pay threshold of each country.

Insulin detemir could be considered cost-effective in Sweden, the UK and the USA, but not in Canada (31–33). Insulin glargine could also be considered cost-effective in Switzerland and the UK but not in Canada (34, 35). A study done in Malaysia showed that insulin glargine and insulin detemir were cost-effective (13). However, this study is different from ours, as its aim was to measure the cost effectiveness of long-acting insulin analogues in comparison to NPH insulin in insulin naïve T2DM patients. In addition, it used the UKPDS-Outcome Model version 2.0 (UKPDS-OM2) to evaluate the cost and consequences of diabetes-related complications.

Novomix 30 could be considered cost-effective in the USA, China and South Korea, while insulin aspart could be considered cost-effective in Canada, Sweden, Spain and Italy but not in Poland (36–39). Insulin lispro was found to be dominant in the UK. It was also associated with reductions in the annual costs of diabetes in Spain, as it reduced the frequency of severe hypoglycaemia (40, 41).

The drug costs were higher in the insulin analogue group than in the conventional human insulin group, but this was partly offset by reduced complication costs. However, another study was done to compare the opposite—the effect of changing from analogue insulin back to human insulin. In 2019, Luo et al. (42) found that encouraging diabetes patients to switch from analogue to human insulin was associated with a small increase in population-level HbA1c but a dramatic reduction in expenditure costs for insulin. This intervention was not associated with any changes in patients’ rates of experiencing serious hypoglycaemia or hyperglycaemia.

## Conclusion

In conclusion, there were no clinically significant improvements in patients’ HbA1c and FBS after the intensification from human insulin to analogue insulin, despite the extra cost spent. However, this study did not take into consideration other factors, such as hypoglycaemia episodes, reduction in hospitalisation and diabetes-related mortality rates. Hence, for physicians to maximise the benefits of insulin analogues at the most optimal cost expenditures, it is vital for them to choose the right group of patients to receive these analogues.

### Strengths, Limitations and Recommendations

The main strength of this study is that it is the first study in Malaysia to analyse the cost effectiveness of human insulin intensification to analogue insulin for a period of 3 months. This study draws attention to the amount of extra costs patients have after changing their prescriptions from human to analogue insulin while also evaluating the immediate effects of such a change on Hba1c and FBS for at least 3 months. This study evaluated the insulin use practices in our hospital. Hopefully, the data we have collected will help clinicians effectively utilise insulin analogues in the treatment of T2DM patients, where our healthcare resources are limited.

Nevertheless, this study has a few limitations. First, the sample size was too small for the data to be applicable to a general diabetes population. We also used a moderate effect size for the sample size calculation, as we anticipated encountering a high rate of missing data due to challenges related to tracing patients’ medical records. Second, due to the retrospective design of this study, data was collected from patients’ medical records. There was a lot of missing data pertaining to factors such as patients’ diet, body weight, compliance to medication and lifestyle. These confounding factors might have affected the results of the study. In addition, we were unable to find justifications for why patients changed from human insulin to analogue insulin due to incomplete documentation in their medical records. However, we found that the mean HbA1c prior to the intensification from human insulin to analogue insulin was high or uncontrolled in all of the patients. Hence, it was assumed that the likeliest reason for their intensifications was poorly controlled T2DM. Another limitation of this study is that we did not include data on the occurrence of hypoglycaemia and its impact on patients’ quality of life. Generally, these are the two main reasons for the intensification of insulin treatment, apart from poor glycaemic control. Lastly, in this study, a cost-effective analysis was not done to correctly measure the pre- and post-intensification of glycaemic control with human and analogue insulin.

A prospective study with a larger sample size and a small effect size should be conducted to further validate the results of this study. This study should include an assessment of patients’ hypoglycaemic episodes, medication adherence and quality of life, as well as whether they have been hospitalised due to severe hypoglycaemia. Follow-up studies should also be done to evaluate patients’ adherence to medication, hypoglycaemia episodes and satisfaction with analogue insulin. These studies should also incorporate a more detailed cost/economic analysis.

## Figures and Tables

**Table 1:** Background characteristics of the patients (*n* = 163)

Variables	Insulin regime				

	Basal (*n* = 51)	Premixed (*n* = 72)	Basal bolus (*n* = 16)	Bolus (*n* = 24)	Total (*n* = 163)
Gender					
Male	25 (49.0)	31 (43.1)	9 (56.3)	15 (62.5)	80 (49.1)
Female	26 (51.0)	41 (56.9)	7 (43.7)	9 (37.5)	83 (50.9)
Age (years old)					
21–40	3 (5.8)	1 (1.4)	2 (12.5)	2 (8.3)	8 (4.9)
41–60	27 (52.9)	37 (51.4)	6 (37.5)	15 (62.5)	85 (52.1)
61–80	21 (41.2)	34 (47.2)	8 (50.0)	7 (29.2)	70 (42.9)

Note: Data presented as number (%)

**Table 2:** Differences in HbA1c and FBS before and after intensification from human insulin to analogue insulin (*n* = 163)

Variables	Insulin type		Mean difference (95% CI)	*t*-statistic (df)	*P*-value

	Human insulin	Analogue insulin			
HbA1c (%)	10.13 (2.00)	9.67 (2.02)	−0.45 (−0.67, −0.24)	−4.164 (162)	< 0.001*
FBS (mmol/L)	10.21 (4.17)	9.25 (4.10)	−0.96 (−1.62, −0.31)	−2.891 (162)	0.004*

Notes: Data presented as mean (SD);

* paired *t*-test;

CI = confidence interval; df = degree of freedom; HbA1c = haemoglobin A1c; FBS = fasting blood sugar

**Table 3:** Differences in HbA1c and FBS for basal, premixed, basal bolus and bolus between human insulin and analogue insulin (*n* = 163)

Variables	Insulin type		Mean difference (95% CI)	*t*-statistic (df)	*P*-value

	Human insulin	Analogue insulin			
HbA1c (%)					
Basal	9.72 (1.83)	9.61 (1.89)	−0.11 (−0.47, −0.25)	−0.618 (50)	0.540
Premixed	10.14 (2.06)	9.55 (2.05)	−0.59 (−0.92, −0.26)	−3.540 (71)	0.001*
Basal bolus	10.09 (1.84)	9.69 (2.10)	−0.40 (−1.05, −0.25)	−1.319 (15)	0.207
Bolus	10.98 (2.07)	10.17 (2.20)	−0.81 (−1.48, −0.14)	−2.502 (23)	0.020*
FBS (mmol/L)					
Basal	10.23 (3.67)	8.81 (3.58)	−1.43 (−2.58, −0.28)	−2.491 (50)	0.016*
Premixed	9.74 (4.09)	9.10 (4.02)	−0.65 (−1.62, 0.32)	−1.332 (71)	0.187
Basal bolus	11.72 (4.83)	9.84 (4.94)	−1.88 (−4.54, 0.79)	−1.500 (15)	0.154
Bolus	10.55 (4.88)	10.22 (4.77)	−0.33 (−2.26, 1.60)	−0.353 (23)	0.727

Notes: Data presented as mean (SD);

* paired *t*-test;

CI = confidence interval; df = degree of freedom; HbA1c = haemoglobin A1c; FBS = fasting blood sugar

**Table 4:** Differences in three months cost between human insulin and analogue insulin (*n* = 163)

Variables	Total cost for 3 months (RM)	Mean difference (95% CI)	*t*-statistic (df)	*P*-value
Human insulin	104.70 (71.29)	292.85 (265.79, 319.91)	21.372 (162)	< 0.001*
Analogue insulin	397.55 (242.10)			

Notes: Data presented as mean (SD);

* paired *t*-test;

CI = confidence interval; df = degree of freedom
